# Substance use is related to differential activation and connectivity for social relative to monetary rewards

**DOI:** 10.1101/2023.01.17.524305

**Published:** 2023-01-19

**Authors:** James B. Wyngaarden, Camille R. Johnston, Daniel Sazhin, Jeff B. Dennison, Ori Zaff, Dominic Fareri, Michael McCloskey, Lauren B. Alloy, David V. Smith, Johanna M. Jarcho

**Affiliations:** 1 Department of Psychology & Neuroscience, Temple University, Philadelphia, PA, USA; 2 Derner School of Psychology, Adelphi University, Garden City, NY, USA

## Abstract

Substance use (SU) has been linked to alterations in reward processing in the ventral striatum (VS). However, less is known about how SU relates to striatal activation and connectivity during social rewards (e.g., positive peer feedback). In this pre-registered study, we hypothesized that SU would be associated with activation and functional connectivity of the VS during receipt of social rewards. Participants (N=44) underwent two fMRI tasks to isolate neural response to social and monetary rewards. The tasks involved choosing between two stimuli: either two purported peers, with the goal of identifying the peer who liked the participant (social); or two doors, with the goal of identifying the door containing a $0.50 prize (monetary). We predicted that VS activation in response to social rewards would be correlated with SU, independent of reward sensitivity (RS); however, an exploratory whole-brain analysis revealed SU was related to activation in the temporoparietal junction instead. Moreover, results showed that aberrant RS blunts the relationship between SU and striatal activation during receipt of rewards, regardless of their domain. Psychophysiological interaction (PPI) analysis demonstrated that SU was associated with decreased connectivity between the VS and dorsomedial prefrontal cortex for social rewards, independent of RS. Exploratory analyses further revealed that RS is associated with increased connectivity between the VS and ventromedial prefrontal cortex during social rewards. Taken together, these findings shed light on the relationships between potential risk factors for developing substance use disorder.

## Introduction

Substance Use Disorder (SUD) is a major health concern affecting over 20 million Americans (Avena et al., 2021). Between April 2020 and 2021, 92,000 people died from drug overdose—the highest death toll during a 12-month period on record (NCDAS, 2022). One common risk factor for developing SUD may be abnormalities in reward processing. For example, individuals with a hyposensitive reward system may be predisposed toward using psychoactive substances to compensate and up-regulate their reward systems (Blum et al., 1996). However, people with a hypersensitive reward system tend to be more impulsive and risk-seeking, factors that also may contribute to risky substance use (SU; Nusslock & Alloy, 2017). These seemingly conflicting observations suggest that disparate mechanisms may lead to a common clinical outcome.

Reward sensitivity (RS) is a self-reported predisposition to seek rewarding substances and experiences (e.g., food, sex, etc.; Volkow et al., 2010). Neuroimaging studies have shown that RS relates to activity in the ventral striatum (VS) and medial prefrontal cortex (Beaver et al., 2006; Simon et al., 2010; Sripada et al., 2011; Pujara et al., 2016). Importantly, abnormalities in reward processing may underlie risky SU (e.g., Bart et al., 2021). One theory of risk for SUD argues that repeated SU over time blunts reward-related activation in the VS and ventromedial prefrontal cortex (vmPFC; Beck at al., 2009; Goldstein & Volkow, 2011). Other evidence suggests SU is associated with hypersensitivity to rewards: for example, higher behavioral approach scores are associated with alcohol use (Franken & Muris, 2006), cocaine addiction (Balconi & Finocchiaro, 2015), and nicotine dependence (Lawn et al., 2015), and adolescents with higher RS scores are more likely to develop future SUD (Alloy et al., 2009; Ivanov et al., 2008; Genovese & Wallace, 2007). Thus, different mechanisms may underlie risk for SUD. Despite these insights, understanding of the brain-based relationship between SU and RS may be limited by a reliance on narrowly focused reward paradigms (e.g., only evaluating monetary outcomes; e.g., Dennison et al., 2022; Bart et al., 2021).

While SU often occurs in a social context (Goncy et al., 2013; Wei et al., 2010; Andrews et al., 2002; Dishion & Owen, 2002) and social stressors, such as peer rejection, commonly precipitate consumption (Nepon et al., 2021; Milivojevic & Sinha, 2018), relationships between SU and RS in the context of *social* rewards are surprisingly understudied. The few studies to test relations between social reward and brain function have shown that SU is associated with decreased activation in the striatum during social rewards (e.g., Jarcho et al., 2022; Zimmermann et al. 2019). However, even fewer studies include brain responses to both social and monetary rewards, which are necessary to make claims about the specificity of effects. Additionally, associations between SU and VS-based connectivity during social reward receipt remain relatively unexplored. Social rewards do elicit VS activation (Izuma et al., 2008), and recent meta-analyses show that processing social information is related to engagement of the dorsal and ventral medial prefrontal cortex (dmPFC, vmPFC), posterior cingulate cortex (PCC), right fusiform face area (FFA), and bilateral amygdala (Tso et al., 2018; Feng et al., 2021; Martins et al., 2021). Therefore, such regions may be promising targets for VS connectivity during receipt of social rewards (e.g., Sazhin et al., 2020).

We leveraged well-matched social and monetary reward tasks (Distefano et al., 2018; Nelson & Jarcho, 2021; Quarmley et al., 2019) to investigate associations between risky SU, RS, and brain activation and connectivity. First, we aimed to quantify the relationship between SU, RS, and VS activation during social compared to monetary rewards. We hypothesized that individuals with higher levels of SU would show exaggerated VS responses to social-vs-monetary rewards, independent of RS. Second, we aimed to examine the relationship between SU, RS, and ventral striatal connectivity during social-vs-monetary rewards. We hypothesized that individuals with higher levels of SU would show enhanced connectivity between the VS and regions modulated by social information, independent of RS.

## Materials and Methods

### Participants

Although the pre-registration (https://aspredicted.org/blind.php?x=JNH_EGK) describes our goal to collect data from 100 participants (18–22), we ultimately studied 59 participants due to constraints imposed by the COVID-19 pandemic. Using pre-registered criteria, fifteen of the 59 participants who completed the study were excluded from analyses due either to failure to respond during behavioral tasks (>20% missing responses; N=4), incomplete data (N=5; failure to complete survey data or missing behavioral data due to technical issues), or poor image quality (N=6). Image quality was defined using the fd_mean and tSNR values from MRIQC (Esteban et al., 2017). Participants were excluded for fd_mean values greater than 1.5 times the interquartile range, or for tSNR values below the lower bound of 1.5 times the interquartile range, per the distribution from neuroimaging data of otherwise eligible participants. This resulted in a final sample of 44 participants (mean age: 20.45 years, SD: 1.89 years; 22.7% male; 57% white, 34% Asian, 9% other—2 Black/African American, 1 Black and white, 1 Indian).

Participants were recruited via the Temple University Psychology and Neuroscience Department participant pool, and from the surrounding community via flyers and online advertisements. Participants were paid $25 per hour for fMRI and $15 per hour for behavioral tasks, and received bonuses based on their decisions on other neuroeconomic tasks (not reported here), resulting in a total payment of $140 to $155. In addition, participants recruited from the university pool also received research credit for their participation.

### Procedure

All methods were approved by the Temple University IRB. Prospective participants were identified based on their responses to an online screener questionnaire, which assessed RS using the Behavioral Activation Subscale (BAS; Carver & White, 1994) and the Sensitivity to Reward subscale (SR; Torrubia & Tobeña, 1984). A sum was calculated for each subscale. Sums were assigned to a quintile that reflected low to high levels of RS across the distribution of possible scores. Using methods consistent with our prior work (e.g., Alloy et al., 2009) designed to increase confidence that participants were truthful, attentive, and not responding at random, only participants with scores within +/−1 quintile on both subscales were eligible for the study (no exclusions were made based on this criteria). SU information also was collected on the online screener questionnaire via the Alcohol Use Disorders Identification Test (AUDIT; Babor et al., 1992) and the Drug Use Identification Test (DUDIT; Berman et al., 2002). At the in-person visit, we confirmed that eligible participants were free of major psychiatric or neurologic illness and MRI contraindications. Breathalyzer tests, urine drug screens, and (for females) urine pregnancy tests were also collected to confirm the absence of alcohol, illicit drugs, and pregnancy prior to participants completing fMRI.

Participants were told that they would complete a social evaluation task with peers who had already completed the study. Prior to the main scan appointment, our team took a photograph of the participant that was purportedly uploaded to a study database. Participants believed that once this photograph was uploaded, peers would receive a text message on their cell phone asking them to view the photo and indicate whether they thought they would ‘like’ or ‘dislike’ the participant. Participants were told that at their main scan appointment several days later (after enough time had elapsed for the purported peers to have rated their photo), they would be asked to guess which peers ‘liked’ and ‘disliked’ them. Participants were also told that they would be completing monetary guessing tasks.

#### fMRI-Based Tasks.

**Figure 1.**
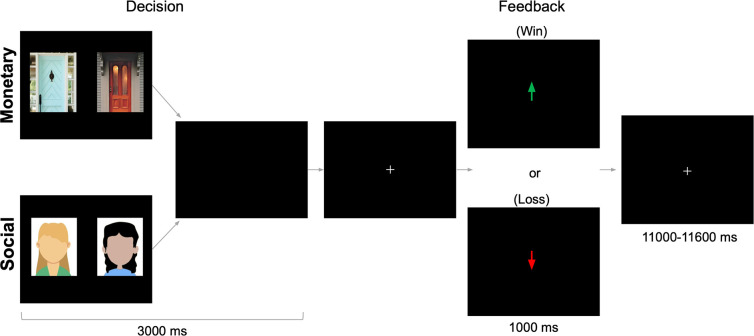
fMRI-based monetary and social reward tasks. On each trial, participants choose either between two doors (monetary task) or the faces of two peers (social task) in search of a reward (note that images shown here are cartoons for illustrative purposes only since journal policies prohibit showing actual photographs). After a brief interval, they receive feedback: an upward arrow indicating a win (monetary task=$0.50 gain; social task=positive peer feedback) or a downward arrow indicating a loss (monetary task=$0.25 loss; social task=negative peer feedback).

The monetary and social reward tasks (Distefano et al., 2018; Nelson & Jarcho, 2021; Quarmley et al., 2019) were administered using PsychoPy (Peirce et al., 2019). As depicted in Figure 1, There were two conditions (monetary, social) that were presented in a counterbalanced order. Each condition included a single run of 60 trials. Half of all trials resulted in reward feedback and the other half resulted in loss feedback. Each task collected 292 functional volumes. Trials were separated by a variable duration intertrial interval (1,100–11,600 ms; M = 3,500ms).

#### Monetary Reward Task.

Participants were instructed to choose the door behind which there was a $0.50 prize, and were told that if they chose incorrectly, they would receive a $0.25 loss. Each trial began with the presentation of two doors. Participants used a button box to select either the left or right door on the screen. This “decision phase” totaled 3,000 ms. Specifically, when a button was pressed, a white border appeared around the selected door for 500 ms before stimulus offset. After stimulus offset, a blank screen appeared for the remainder of the 3,000 ms decision phase. A fixation cross then was presented (540 ms) before participants received feedback about the accuracy of their choice (1,000 ms). On reward trials, feedback was a green arrow pointing upwards, meaning the participant correctly selected the winning door. On loss trials, feedback was a red arrow pointing downwards, meaning the participant incorrectly selected the losing door. On trials where the participant failed to respond, feedback was randomly selected. Note that the first 12 participants, collected prior to the pandemic, saw pairs of fractals during this task. After data collection resumed, pictures of doors were used instead to more closely match with the realistic features of peer images. This change did not impact response time following monetary rewards vs. losses (Welch Two Sample t-test; t = 0.75, p = 0.46) nor striatal response to monetary rewards vs. losses (Welch Two Sample t-test; t = 0.13, p = 0.90). We therefore collapsed across participants with fractal vs. door stimuli.

#### Social Reward Task.

The social task was identical to the monetary task, except images of gender-matched (i.e., two female faces or two male faces) and age-matched peers were presented instead of doors. The social reward task consisted of 120 images compiled from multiple sources (internet databases of non-copyrighted images of college-aged individuals). The pictures of purported peers had positive facial expressions, were cropped so that individuals were pictured from their shoulders up and were edited to have an identical solid gray background. Smiling faces were used because they are common in social reward tasks (Richards et al., 2013; Jarcho et al., 2015; Distefano et al., 2018), and are subject to less misinterpretation than neutral faces (Rapee and Heimberg, 1997; Davis et al., 2016). Images were constrained to a standard size (aspect ratio: 11.2 width, 17.14 height). There were an equal number of trials with male and female peers across the reward and loss conditions (30 pairs each, 60 total). Participants were instructed to choose the peer that liked them based on the photograph of the participant. On reward trials, feedback was a green arrow pointing upwards, meaning the participant correctly selected the person who said they would like the participant. On loss trials, feedback was a red arrow pointing downwards, meaning they incorrectly selected the person who said they would dislike the participant. On trials where the participant failed to respond, feedback was randomly selected.

### Individual Difference Measures

#### Reward Sensitivity.

RS was defined by a composite score consisting of the sum of the z-scores for the Behavioral Activation Scale (BAS; Carver & White, 1994) and the Reward subscale (RS) of the Sensitivity to Punishment/Sensitivity to Reward Questionnaire (Torrubia et al., 2001). The BAS is a 20-item self-report questionnaire that aims to measure sensitivity to appetitive motives (e.g., “I go out of my way to get things I want”). The SR is a 24-item self-report measure aimed at more context-specific items. The total BAS reward scale and the Sensitivity to Reward (SR) subscale of SPSRQ are reliable and valid measures of RS (Alloy et al., 2006; Alloy et al., 2012).

To properly control for the effect of RS, we focused on both linear and nonlinear associations between brain responses and RS. However, assessing nonlinear associations with RS (i.e., aberrant RS) by taking the 2^nd^-order polynomial expansion will overweight the tails of the distribution in our analyses (Buchel et al., 1998). To avoid this, we first normalized the values by converting to deciles, where each decile had approximately the same number of participants (cf. Winsorizing). Although this strategy was not described in our pre-registration, we believe it is an important deviation because it ensures that our analyses can control for both linear and nonlinear (e.g., U-shaped and inverted U-shaped patterns between brain responses and RS) that are not driven by extreme values in the tails of the distribution.

#### Substance Use.

SU was defined by a composite score consisting of the sum of the z-scores for the Alcohol Use Disorders Identification Test (AUDIT; Babor et al., 1992) and the Drug Use Identification Test (DUDIT; Berman et al., 2002). The AUDIT is a 10-item self-report measure that assesses frequency (e.g., “How often do you have a drink containing alcohol?”) and disruptiveness (e.g., “How often during the last year have you failed to do what was normally expected of you because of drinking?”) of alcohol use. Scores greater than 15 are categorized as high risk for alcohol dependence (Babor et al., 2001). No participant scored in the high-risk category. The DUDIT is an 11-item self-report measure that assesses frequency and disruptiveness of non-alcoholic drug use, containing references to a wide array of substances, including marijuana, cocaine, and others. Previous research has established scores greater than 24 as a threshold for high-risk for dependence (Berman et al., 2005). Only one participant scored in the high-risk category (score=26). We used a sum of z-scores for AUDIT and DUDIT because we did not have hypotheses differentiating between alcohol use and other drug use.

### Neuroimaging Data Acquisition

Functional images were acquired using a 3.0 Tesla Siemens PRISMA MRI scanner and a 20-channel head coil. Bold Oxygenation Level-Dependent (BOLD) sensitive functional images were acquired using a simultaneous multislice(multi-band factor = 2) gradient echo-planar imaging (EPI) sequence (240 mm in FOV, TR = 1,750 ms, TE = 29 ms, voxel size of 3.0 × 3.0 × 3.0 mm^3^, flip angle = 74°, interleaved slice acquisition, with 52 axial slices). Each run included 292 functional volumes. We also collected single-band reference images with each functional run of multi-band data to improve motion correction and registration. To facilitate anatomical localization and co-registration of functional data, a high-resolution structural scan was acquired (sagittal plane) with a T1-weighted magnetization=prepared rapid acquisition gradient echo (MPRAGE) sequence (224 mm in FOV, TR = 2,400 ms, TE = 2.17 ms, voxel size of 1.0 × 1.0 × 1.0 mm^3^, flip angle 8°). In addition, we also collected a B0 fieldmap to unwarp and undistort functional images (TR: 645 ms; TE1: 4.92 ms; TE2: 7.38 ms; matrix 74×74; voxel size: 2.97×2.97×2.80 mm; 58 slices, with 15% gap; flip angle: 60°).

### Preprocessing of Neuroimaging Data

Neuroimaging data were converted to the Brain Imaging Data Structure (BIDS) using HeuDiConv version 0.9.0 (Halchenko et al., 2019). Results included in this manuscript come from preprocessing performed using fMRIPrep 20.2.3 (Esteban et al., 2018a, 2018b), which is based on Nipype 1.4.2 (Gorgolewski et al., 2011, 2018). The details described below are adapted from the fMRIPrep preprocessing details; extraneous details were omitted for clarity.

#### Anatomical data preprocessing.

The T1-weighted (T1w) image was corrected for intensity non-uniformity (INU) with ‘N4BiasFieldCorrection’, distributed with ANTs 2.3.3, and used as T1w-reference throughout the workflow. The T1w-reference was then skull-stripped with a *Nipype* implementation of the ‘antsBrainExtraction.sh’ workflow (from ANTs), using OASIS30ANTs as target template. Brain tissue segmentation of cerebrospinal fluid (CSF), white-matter (WM), and gray-matter (GM) was performed on the brain-extracted T1w using ‘fast’ (FSL 5.0.9). Volume-based spatial normalization to one standard space (MNI152NLin2009cAsym) was performed through nonlinear registration with ‘antsRegistration’ (ANTs 2.3.3), using brain-extracted versions of both T1w reference and the T1w template. The following template was selected for spatial normalization: *ICBM 152 Nonlinear Asymmetrical template version 2009c* (TemplateFlow ID: MNI152NLin2009cAsym)

#### Functional data preprocessing.

For each of the BOLD runs per subject, the following preprocessing steps were performed. First, a reference volume and its skull-stripped version were generated by aligning and averaging 1 single-band references (SBRefs). A B0-nonuniformity map (or *fieldmap*) was estimated based on a phase-difference map calculated with a dual-echo GRE (gradient-recall echo) sequence, processed with a custom workflow of *SDCFlows* inspired by the ‘epidewarp.fsl’ script (http://www.nmr.mgh.harvard.edu/~greve/fbirn/b0/epidewarp.fsl) and further improvements in HCP Pipelines. The *fieldmap* was then co-registered to the target EPI (echo-planar imaging) reference run and converted to a displacements field map (amenable to registration tools such as ANTs) with FSL’s ‘fugue’ and other *SDCflows* tools. Based on the estimated susceptibility distortion, a corrected EPI (echo-planar imaging) reference was calculated for a more accurate co-registration with the anatomical reference. The BOLD reference was then co-registered to the T1w reference using ‘flirt’ (FSL 5.0.9) with the boundary-based registration cost-function. Co-registration was configured with nine degrees of freedom to account for distortions remaining in the BOLD reference. Head-motion parameters with respect to the BOLD reference (transformation matrices, and six corresponding rotation and translation parameters) are estimated before any spatiotemporal filtering using ‘mcflirt’.

BOLD runs were slice-time corrected using ‘3dTshift’ from AFNI 20160207. First, a reference volume and its skull-stripped version were generated using a custom methodology of *fMRIPrep*. The BOLD time-series (including slice-timing correction when applied) were resampled onto their original, native space by applying a single, composite transform to correct for head-motion and susceptibility distortions. These resampled BOLD time-series will be referred to as *preprocessed BOLD in original space*, or just *preprocessed BOLD*. The BOLD time-series were resampled into standard space, generating a *preprocessed BOLD run in MNI152NLin2009cAsym space*. First, a reference volume and its skull-stripped version were generated using a custom methodology of *fMRIPrep*. Several confounding time-series were calculated based on the *preprocessed BOLD,* notably including framewise displacement (FD).

Additionally, a set of physiological regressors were extracted to allow for component-based noise correction (*CompCor*). Principal components are estimated after high-pass filtering the *preprocessed BOLD* time-series (using a discrete cosine filter with 128s cut-off) for anatomical component correction (aCompCor). For aCompCor, three probabilistic masks (CSF, WM and combined CSF+WM) are generated in anatomical space. The implementation differs from that of Behzadi et al. in that instead of eroding the masks by 2 pixels on BOLD space, the aCompCor masks are subtracted from a mask of pixels that likely contain a volume fraction of GM. This mask is obtained by thresholding the corresponding partial volume map at 0.05, and it ensures components are not extracted from voxels containing a minimal fraction of GM. Finally, these masks are resampled into BOLD space and binarized by thresholding at 0.99 (as in the original implementation). Components are also calculated separately within the WM and CSF masks. For each CompCor decomposition, the *k* components with the largest singular values are retained, such that the retained components’ time series are sufficient to explain 50 percent of variance across the nuisance mask (CSF, WM, combined, or temporal). The remaining components are dropped from consideration. The head-motion estimates calculated in the correction step were also placed within the corresponding confounds file. All resamplings can be performed with *a single interpolation step* by composing all the pertinent transformations (i.e., head-motion transform matrices, susceptibility distortion correction when available, and co-registrations to anatomical and output spaces). Gridded (volumetric) resamplings were performed using ‘antsApplyTransforms’ (ANTs), configured with Lanczos interpolation to minimize the smoothing effects of other kernels.

Many internal operations of *fMRIPrep* use *Nilearn* 0.6.2, mostly within the functional processing workflow. For more details of the pipeline, see the section corresponding to workflows in *fMRIPrep*’s documentation (https://fmriprep.readthedocs.io/en/latest/workflows.html).

Further, we applied spatial smoothing with a 5mm full-width at half-maximum (FWHM) Gaussian kernel using FEAT (FMRI Expert Analysis Tool) Version 6.00, part of FSL (FMRIB’s Software Library, www.fmrib.ox.ac.uk/fsl). Non-brain removal using BET (Smith, 2002) and grand mean intensity normalization of the entire 4D dataset by a single multiplicative factor were also applied.

### Neuroimaging Analyses

#### Individual Level Analyses.

Neuroimaging analyses used FSL version 6.0.4 (Smith et al., 2004; Jenkinson et al., 2012). We specifically focused on two types of analyses (activation and connectivity) to investigate how social rewards and SU were associated with BOLD responses, independent of linear or quadratic expressions of RS (RS). Importantly, exploratory analyses also examined RS as a covariate of interest to assess the impact of RS in the case that our main hypotheses were not supported. Both used individual level general linear models with local autocorrelation (Woolrich et al., 2001).

The first model focused on the brain activation evoked during the feedback phase of each reward task (i.e., monetary and social) and used a total of four task regressors. Two regressors of interest included reward and loss feedback; (duration = 1,000 ms). Two regressors of no interest included the decision phase (duration = 3,000 ms) and trials with missed responses within the decision phase (i.e., failures to respond; duration = 3,000 ms).

Our second type of model focused on task-dependent connectivity with VS as it related to the varying types of feedback in the task (i.e., reward vs. loss). To estimate the changes in connectivity between feedback types, we used psychophysiological interaction (PPI) analysis (Friston et al., 1997; O’Reilly et al., 2012), which can reveal consistent and specific task-dependent changes in connectivity (Smith et al., 2016; Smith & Delgado, 2017). We focused on feedback-dependent changes in connectivity with the bilateral VS (Oxford-GSK-Imanova atlas; Tziortzi et al., 2011). The connectivity model used a total of nine regressors. The first four were identical to the four described in the activation model (i.e., reward, loss, decision, and missed trials). A fifth regressor consisted of the average timecourse of activation from the VS seed region, also referred to as the physiological regressor. Finally, four additional regressors were added to the PPI model, each corresponding to the interaction between the physiological regressor and the four original regressors.

Both activation and connectivity models included additional regressors of no interest that controlled for six motion parameters (rotations and translations), the first six aCompCor components explaining the most variance, non-steady state volumes, and framewise displacement (FD) across time. Finally, high-pass filtering (128s cut-off) was achieved using a set of discrete cosine basis functions.

### Hypothesis 1

#### ROI Group Level Analysis.

Hypothesis 1 seeks to test whether individuals reporting higher levels of SU show exaggerated ventral striatal responses to social rewards relative to monetary rewards, independent of expressions of RS. To control for potential relations with RS, both linear (i.e., greater values correspond with greater RS) and quadratic (i.e., greater values correspond with more aberrant RS) measures of RS were included as covariates of no interest. For each participant, activation in the VS seed described above was extracted using AFNI’s 3dROIstats. We used FSL’s PALM (Winkler et al., 2014; Alberton et al., 2020) to conduct a linear regression for the difference in striatal BOLD for social reward processing (see Table 1 for notation) that was regressed onto a model of SU including additional covariates for RS (first- and second-order measures), the SUxRS and SUxRS^2^ interactions, and nuisance regressors (tSNR and motion; seven covariates total). Given that social and monetary tasks were administered separately, it is critical that we account for differences in the quality of confounds like temporal signal-to-noise ratio (tSNR) and framewise displacement. To account for these differences, we subtracted the value from the monetary task from the social task.

### Hypothesis 1 Exploratory Analyses

#### ROI Group Level Analysis.

We hypothesized that we would observe a domain × feedback × SU interaction. However, lower order interactions that included task effects (i.e., domain or feedback) were also explored to test the extent to which SU may relate to brain activity as a function of other task features. Exploratory ROI-based analyses also examined relations with RS as a covariate of interest.

#### Whole Brain Group Level Analyses.

We also conducted exploratory whole brain analyses to investigate regions outside of the VS that may be implicated in SU, reward, and social processes. Group-level analyses were conducted using Randomise (Winkler et al., 2014) and focused on social reward processing using the same linear regression model described above. In addition to SU, whole brain exploratory analyses also investigated relations with RS as a covariate of interest.

### Hypothesis 2

#### PPI Target Regions.

**Figure 2.**
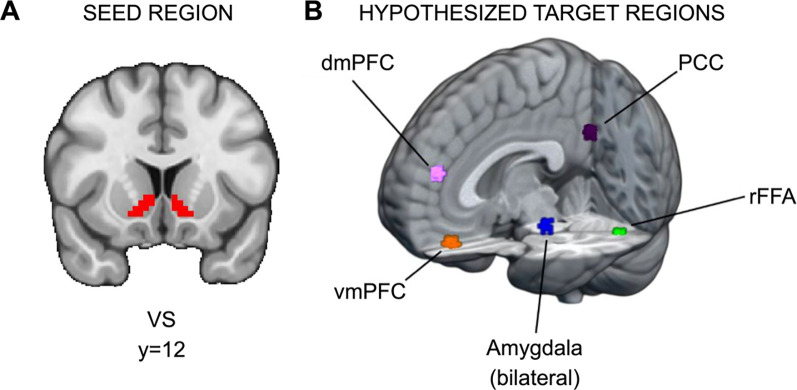
ROIs for PPI analyses. (A) Ventral striatum seed region and (B) hypothesized target regions for PPI analyses (https://identifiers.org/neurovault.collection:13364). Hypothetical target regions related to social information processing in the current dataset were identified via significant clusters in the decision phase. For each target region, a 5mm sphere was drawn around the significant cluster’s peak voxel. The amygdala targets were constrained to voxels within the amygdala as defined by Harvard-Oxford Atlas.

Hypothesis 2 seeks to investigate whether SU, independent of reward sensitivity, is associated with elevated connectivity between the VS and regions implicated in social information processing (e.g., ventromedial prefrontal cortex) during responses to social reward, relative to monetary reward. To this end, we sought to identify brain regions modulated by social information (i.e., stimuli in the social condition) in the current dataset. To isolate those regions, we contrasted brain function during the decision phase between the social task, where participants viewed pictures of peers, and the monetary task, where participants viewed pictures of doors. Significant clusters of activation were found in several regions, and for each potential target region, a 5mm sphere was drawn around the peak voxel in the cluster (see Figure 2 and https://identifiers.org/neurovault.collection:13364). Significant regions were the ventromedial prefrontal cortex (vmPFC; x=33, y=61, z=20), dorsomedial prefrontal cortex (dmPFC; x=34, y=62, z=34), right fusiform face area (rFFA; x=47, y=26, z=20), bilateral amygdala (right, x=39, y=42, z=22; left, x=25, y=42, z=22), and posterior cingulate cortex (PCC; x=33, y=25, z=36). There are two caveats for amygdala: first, for the right amygdala, the cluster extended across multiple regions, so a local maximum within the Harvard-Oxford-defined anatomical region for right amygdala was used instead; second, because amygdala is a smaller anatomical region relative to the other potential targets, after drawing 5mm spheres, both amygdala ROIs were constrained to the Harvard-Oxford anatomical atlas.

#### PPI ROI Analysis.

For each participant, connectivity estimates for the target regions described above (vmPFC, dmPFC, rFFA, bilateral amygdala, and PCC) were extracted using AFNI’s 3dROIstats from individual level analyses that modeled the timecourse of VS as a seed. For each target region, we used FSL’s PALM (Winkler et al., 2014; Alberton et al., 2020) to conduct linear regression models for the difference in functional connectivity between VS seed and target ROIs for social reward processing. This was regressed onto a model of SU including additional covariates for RS (first- and second-order measures), the SU × RS and SU × RS^2^ interactions, and nuisance regressors (tSNR and motion). Specifically, we hypothesized that we would observe a domain × feedback × SU interaction.

### Hypothesis 2 Exploratory Analyses

#### PPI ROI Analysis.

Hypothesis 2 examines the domain × feedback × SU interaction in the hypothesized target regions. However, lower order interactions that included task effects (i.e., domain or feedback) were also explored to test the extent to which SU may relate to connectivity as a function of other task features. In addition to SU, exploratory analyses also investigated relations with RS.

## Results

### Hypothesis 1

#### No Association between Substance Use and Ventral Striatum Activation During Social vs. Monetary Rewards.

Our first goal was to examine whether higher levels of SU were associated with exaggerated ventral striatal response to social relative to monetary rewards, independent of self-reported RS. Inconsistent with our first pre-registered hypothesis, we did not observe a significant association between SU and striatal activation for social vs. monetary rewards (*t*_(36)_=−1.1077, *p*=0.865; full model results in Table 2).

### Hypothesis 1 Exploratory Results

#### Aberrant Reward Sensitivity is Associated with a Diminished Relation between Substance Use and Striatal Activation During Reward Receipt.

**Fig. 3.**
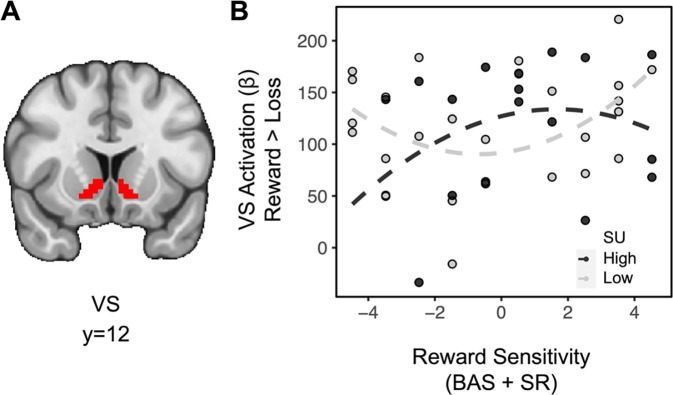
Aberrant reward sensitivity blunts the relationship between substance use and striatal activation during receipt of rewards. (A) Ventral striatum ROI. (B) For individuals with moderate levels of RS (N=26), greater levels of SU are weakly associated with striatal activation for rewards. However, for individuals with aberrant RS (N=18), greater levels of SU are associated with decreased striatal activation for rewards. [Aberrant reward was specified as individuals in the top 2 and bottom 2 deciles of the first-order RS measure (i.e., individuals with positive values for second-order RS when de-meaned)].

Linear regressions for lower order interactions to assess striatal effects related to domain and feedback also were examined to test the extent to which SU and RS may relate to brain activity as a function of other task features. Across all subjects, the difference in striatal BOLD for feedback [(social reward + monetary reward) > (social loss + monetary loss)] showed a significant effect (*t*_(36)_=13.534, *p*<0.001), indicating greater striatal activation for rewards relative to losses (full model results in Table 2). Importantly, there was no difference in striatal activation between domains (e.g., *t*_(36)_=−0.774, *p*=0.779), suggesting that degree of reward processing across paradigms was similar. A linear regression for the difference in striatal BOLD for feedback on the interaction between SU and quadratic (aberrant) RS also revealed a significant effect (*t*_(36)_=2.198, *p*=0.018): among individuals with moderate trait RS, SU is weakly associated with rewarding feedback, however, among individuals with aberrant trait RS, SU is negatively associated with rewarding feedback (Fig. 3). A linear regression for the difference in striatal BOLD for domain [(social reward + social loss) > (monetary reward + monetary loss)] revealed no significant relationships.

#### Substance Use is Associated with Blunted TPJ Responses to Social vs. Monetary Reward.

**Figure 4.**
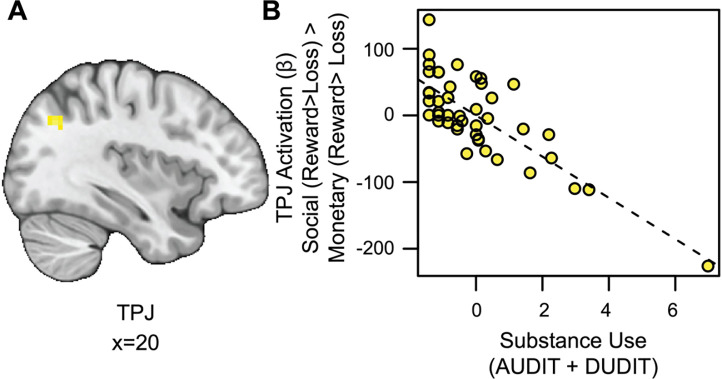
Substance use is associated with blunted TPJ responses to social vs. monetary reward. (A) Cluster-level thresholded activation (Z > 3.1, FWE = 0.05) for social rewards in the temporoparietal junction (TPJ) related to SU (Thresholded: https://identifiers.org/neurovault.image.790569; Unthresholded: https://identifiers.org/neurovault.image.790568). (B) While controlling for linear and quadratic measures of RS: as SU increases, social reward activation in the TPJ (component + residual) decreases.

We also conducted an exploratory whole-brain analysis to investigate the relation between SU and activation for social rewards beyond the VS. This analysis revealed a significant cluster of activation in the temporoparietal junction for the social rewards in relation to SU (TPJ; see Fig. 4A and https://identifiers.org/neurovault.image:790569). Extracting parameter estimates from the TPJ (x=14, y=19, z=38; ke = 56) revealed that as SU increased, activation in the TPJ decreased for social relative to monetary reward (Fig. 4B).

### Hypothesis 2

#### Substance Use is Associated with Reduced Task-Based Connectivity between the VS and dmPFC During Social vs. Monetary Reward.

As described in our pre-registration, we hypothesized that elevated ventral striatal responses to social reward, relative to monetary reward, would be associated with enhanced connectivity with regions modulated by social information, independent of RS. To test this hypothesis, we conducted an ROI-based psychophysiological interaction (PPI) analysis using the VS as our seed and five social regions as targets. Linear regressions for lower order interactions of domain and feedback also were assessed. No significant effects were observed in the amygdala, rFFA, or PCC for our interactions of interest.

**Figure 5.**
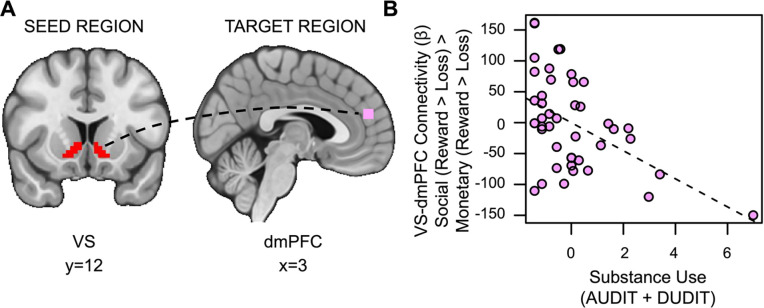
Substance use is associated with decreased VS-dmPFC connectivity for social vs. monetary reward. (A) Ventral striatum seed region and dmPFC target region. (B) As SU increases, connectivity between the ventral striatum and the dorsomedial prefrontal cortex (dmPFC) during receipt of social rewards is reduced.

Contrary to our second hypothesis, we found that VS-dmPFC connectivity for social relative to monetary reward was attenuated in individuals with more severe SU (*t*_(36)_=2.525, *p*=.007, *family-wise-error-corrected p (fwep)*=.0353; full model results in Table 3). As SU increases, connectivity between the VS and dmPFC is reduced (Fig. 5).

#### Hypothesis 2 Exploratory Results

**Figure 6.**
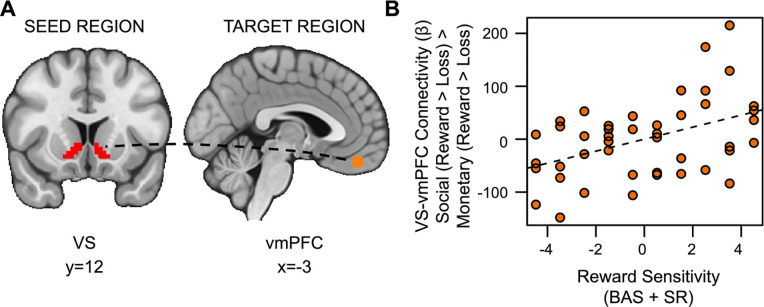
Reward sensitivity is associated with increased VS-vmPFC connectivity for social vs. monetary reward. (A) Ventral striatum seed region and vmPFC target region. (B) As reward sensitivity increases, connectivity between the ventral striatum and the ventromedial prefrontal cortex (vmPFC) during receipt of social rewards is enhanced.

Also inconsistent with our hypotheses, social rewards showed enhanced connectivity between the VS and vmPFC in relation to RS (*t*_(36)_=2.528, *p*=.007, *fwep*=.0356; full model results in Table 4). As RS increases, functional connectivity between the VS and vmPFC is enhanced (Fig. 6).

## Discussion

We leveraged well-matched social and monetary reward tasks (Distefano et al., 2018; Nelson & Jarcho, 2021; Quarmley et al., 2019) to investigate associations between SU, RS, and brain activation. Although we hypothesized that SU would be associated with VS activation for social rewards independent of RS, we did not find support for this hypothesis. However, exploratory whole-brain analyses did find this effect in the TPJ, such that SU was associated with decreased TPJ activation for social vs. monetary rewards while controlling for RS. Moreover, exploratory investigation of lower order effects showed that aberrant RS blunts the relationship between SU and VS activation during receipt of domain-general rewards. Contrary to our second hypothesis, our findings show that SU is associated with decreased VS-dmPFC connectivity for social vs. monetary rewards. Additionally, exploratory analyses showed that RS is associated with increased VS-vmPFC connectivity for social vs. monetary rewards. Taken together, these findings suggest that sub-clinical SU is associated with blunted response (TPJ) and diminished corticostriatal connectivity (VS-dmPFC) for social relative to monetary rewards, while underscoring the nuanced role of trait RS.

Very few studies have investigated the impact of SU on processing social rewards in particular (see Beard et al., 2022). However, our finding that SU is associated with blunted brain activation (TPJ) and connectivity (VS-dmPFC) for social compared to monetary rewards is in line with the handful of studies. For example, Zimmermann et al. (2019) found that cannabis users showed less activity in the striatum during interpersonal touch from a female experimenter, while non-users showed more striatal activity. Further, Li et al. (2021) showed that binge drinkers had decreased neural responses in the anterior medial orbitofrontal cortex and precuneus when viewing social interactions of abstract shapes, compared to viewing random movement. Finally, a recent study from our research group (Jarcho et al., 2022), completed after preregistration for the current work, found that greater substance abuse behavior in adolescents was related to decreased right VS response to social rewards (peer ‘like’ feedback). Given that SU often occurs in a social context (Goncy et al., 2013; Wei et al., 2010; Andrews et al., 2002; Dishion & Owen, 2002) and social stressors commonly precipitate consumption (Nepon et al., 2021; Milivojevic & Sinha, 2018), these domain-specific effects are worth investigating further.

Our findings also provide evidence suggesting that SU and RS are associated with altered connectivity in regions that support social cognition. SU was related to decreased VS-dmPFC connectivity for social rewards, potentially suggesting that increased SU is related to altered ability to process social information. Striatal connectivity has been linked to interpreting others’ facial expressions (Kim et al., 2020), and the dmPFC has been implicated in social cognitive functions during receipt of social feedback (e.g., Ferrari et al., 2016) and prediction error during social learning (Joiner et al., 2017; Suzuki et al., 2012). Reduced correspondence of the VS with dmPFC during social reward may suggest an impaired facility for assessing and reacting to feedback from others. Additionally, contrary to our hypothesis, trait RS was positively associated with VS-vmPFC connectivity for social rewards. Increased subjective utility for a given choice after observing others make a similar choice has been linked to the vmPFC (Chung et al., 2015), and functional connectivity between the VS and the vmPFC has been shown to reflect subjective value for social rewards (Smith et al., 2014; Hill et al., 2017; Hare et al., 2010). The current results suggest that higher trait RS is associated with increased functional integration of these regions, perhaps bridging the gap to previous research linking RS to social functions like extraversion (Lucas et al., 2000) and anxiety (Booth & Hasking, 2009) which may center on receiving feedback from peers.

There are several limitations to consider when evaluating these results. A key area for future studies to expand into would be to investigate populations with a wider range of SU. The current sample includes only sub-clinical SU scores. Although this feature of the sample allows us to examine risk factors for SUD, the subtle effects we observe here may differ from observations of a clinical sample. However, a benefit of studying those at risk for SU is that effects observed here are unlikely to be a consequence of long-term use that often occurs in clinical populations. Previous research has shown the association between responses in the nucleus accumbens/VS during reward anticipation and future problematic substance use and relapse (e.g., Büchel et al., 2017; MacNiven et al., 2018). To help close the gap between cause and consequence, longitudinal studies could be used to further examine the relationship between sub-clinical SU and development of SUD. Other features of the sample may impact generalizability. For example, the current sample is comprised of college students, who have lower levels of SUD relative to their non-college peers (despite higher rates of heavy alcohol consumption; Skidmore et al., 2016). Differences in social factors related to SU for college vs. non-college individuals may be relevant to consider as well. Finally, the sample is also relatively small, primarily white (57%) and Asian (34%), and predominantly female (77.3%). SUD is more common in males (Brady & Randall, 1999), although this gap is narrowing (Steingrimsson et al., 2012). As neuroimaging research on sex-based differences in relation to substance use and the brain is mixed (e.g., McHugh et al., 2018), further work is needed in larger samples to test for these important biological differences.

Despite these limitations, the present results demonstrate that varying levels of problematic SU in a sub-clinical population is associated with variations in activation and task-based connectivity in regions implicated in social processing while experiencing social rewards. Moreover, we show that trait RS is an important factor when individuals experience social feedback. Although SUD is a complicated issue, our findings help characterize the important roles in how social factors and aberrant reward sensitivity are related to problematic SU. These results may contribute to our understanding of how to identify and reduce instances of SUD in the future.

## Figures and Tables

**Table 1.** Terms for model contrasts.

Term	Effect	Contrast

Social reward processing	Domain × Feedback	Social (Reward > Loss) > Monetary (Reward > Loss)
Reward processing	Feedback	(Social Reward + Monetary Reward) > (Social Loss + Monetary Loss)
Social processing	Domain	(Social Reward + Social Loss) > (Monetary Reward + Monetary Loss)
Decision phase	Social Stimuli	(Social Decision > Baseline) > (Monetary Decision > Baseline)

**Table 2.** Statistics for regression models of striatal activation.

	Domain × Feedback		Domain		Feedback	

Contrast	*tstat*	*uncp*	*tstat*	*uncp*	*tstat*	*uncp*

Main Effect +	−0.7739	0.7793	0.6244	0.257	13.5344	0.0002***
Main Effect −	0.7739	0.2208	−0.6244	0.7431	−13.5344	0.9999
RS +	−0.1752	0.5725	0.5333	0.2985	1.542	0.0619
RS −	0.1752	0.4276	−0.5333	0.7016	−1.542	0.9382
RS^2^ +	−1.4749	0.9226	0.0187	0.4916	0.3478	0.3601
RS^2^ −	1.4749	0.0775	−0.0187	0.5085	−0.3478	0.64
SU +	−1.1077	0.8649	0.3464	0.3596	−1.1312	0.8693
SU −	1.1077	0.1352	−0.3464	0.6405	1.1312	0.1308
SU × RS +	−0.283	0.6148	−0.0643	0.5298	−0.2062	0.5794
SU × RS −	0.283	0.3853	0.0643	0.4703	0.2062	0.4207
SU × RS^2^ +	0.4043	0.3447	−0.611	0.7281	−2.198	0.9817
SU × RS^2^ −	−0.4043	0.6554	0.611	0.272	2.198	0.0184*

Note: RS=first-order measure of reward sensitivity; RS^2^=second-order measure of reward sensitivity (i.e., emphasizes aberrant RS); SU=substance use.

+=positive relationship with striatal activation; −=negative relationship with striatal activation

* (p<.05)

** (p<.01)

*** (p<.001).

**Table 3.** Statistics for regression models of VS connectivity with dmPFC.

	Domain × Feedback			Domain		
Contrast	*tstat*	*uncp*	*fwep*	*tstat*	*uncp*	*fwep*

Main Effect +	0.4504	0.32	0.7291	1.1788	0.1249	0.4363
Main Effect −	−0.4504	0.6801	0.9672	−1.1788	0.8752	0.9999
RS +	1.7279	0.0467*	0.1757	−0.0671	0.5388	0.959
RS −	−1.7279	0.9534	0.9999	0.0671	0.4613	0.9367
RS^2^ +	−0.3105	0.6242	0.9563	−0.5649	0.7199	0.9929
RS^2^ −	0.3105	0.3759	0.7923	0.5649	0.2802	0.7646
SU +	−2.5251	0.9931	1	0.392	0.3437	0.8307
SU −	2.5251	0.007**	0.0353*	−0.392	0.6564	0.9836
SU × RS +	2.0168	0.025*	0.1017	0.9121	0.1826	0.6021
SU × RS −	−2.0168	0.9751	1	−0.9121	0.8175	0.9993
SU × RS^2^ +	−0.2995	0.6126	0.9521	0.786	0.2162	0.6743
SU × RS^2^ −	0.2995	0.3875	0.801	−0.786	0.7839	0.9984

	Feedback					
Contrast	*tstat*	*uncp*	*fwep*			
	
Main Effect +	−0.2658	0.5976	0.922			
Main Effect −	0.2658	0.4025	0.7813			
RS +	−0.9607	0.8216	0.9924			
RS −	0.9607	0.1785	0.4735			
RS^2^ +	−0.1298	0.5476	0.898			
RS^2^ −	0.1298	0.4525	0.8316			
SU +	2.1169	0.0215*	0.0868			
SU −	−2.1169	0.9786	1			
SU × RS +	0.4564	0.323	0.7135			
SU × RS −	−0.4564	0.6771	0.9586			
SU × RS^2^ +	0.6934	0.2458	0.5965			
SU × RS^2^ −	−0.6934	0.7543	0.9783			
	

Note: RS=first-order measure of reward sensitivity; RS^2^=second-order measure of reward sensitivity (i.e., emphasizes aberrant RS); SU=substance use.

+=positive relationship with VS-dmPFC connectivity; −=negative relationship with VS-dmPFC connectivity

* (p<.05)

** (p<.01)

*** (p<.001).

**Table 4.** Statistics for regression models of VS connectivity with vmPFC.

	Domain × Feedback			Domain		
Contrast	*tstat*	*uncp*	*fwep*	*tstat*	*uncp*	*fwep*

Main Effect +	0.0052	0.5048	0.8795	1.8072	0.0389*	0.1716
Main Effect −	−0.0052	0.4953	0.8891	−1.8072	0.9612	1
RS +	2.5282	0.0074**	0.0356*	0.6809	0.2502	0.7327
RS −	−2.5282	0.9927	1	−0.6809	0.7499	0.9968
RS^2^ +	0.8768	0.192	0.5382	−1.7859	0.9597	1
RS^2^ −	−0.8768	0.8081	0.9943	1.7859	0.0404*	0.1827
SU +	−0.5602	0.7031	0.979	2.2154	0.0153*	0.0778
SU −	0.5602	0.297	0.7057	−2.2154	0.9848	1
SU × RS +	2.1603	0.019*	0.0766	1.463	0.0758	0.3075
SU × RS −	−2.1603	0.9811	1	−1.463	0.9243	1
SU × RS^2^ +	0.8184	0.2105	0.57	−0.4081	0.6579	0.991
SU × RS^2^ −	−0.8184	0.7896	0.9929	0.4081	0.3422	0.8391

	Feedback					
Contrast	*tstat*	*uncp*	*fwep*			
	
Main Effect +	−0.1127	0.5431	0.8886			
Main Effect −	0.1127	0.457	0.8338			
RS +	0.4358	0.3319	0.7108			
RS −	−0.4358	0.6682	0.9587			
RS^2^ +	0.9296	0.1818	0.4796			
RS^2^ −	−0.9296	0.8183	0.9895			
SU +	−0.0513	0.5201	0.8856			
SU −	0.0513	0.48	0.8556			
SU × RS +	−2.2799	0.9847	1			
SU × RS −	2.2799	0.0154*	0.0661			
SU × RS^2^ +	0.0171	0.4936	0.8672			
SU × RS^2^ −	−0.0171	0.5065	0.8705			
	

Note: RS=first-order measure of reward sensitivity; RS^2^=second-order measure of reward sensitivity (i.e., emphasizes aberrant RS); SU=substance use.

+=positive relationship with VS-vmPFC connectivity; −=negative relationship with VS-vmPFC connectivity

* (p<.05)

** (p<.01)

*** (p<.001).

**Table 5.** Summary of reported results.

Hyp	Analysis	Effect	Contrast	*tstat*	*p*	*corr-p*

H1	Domain × Feedback	VS act	SU +	−1.108	0.87	-
H1: ExA	Feedback	VS act	SU × RS^2^ −	2.198	<.05*	-
H1: ExB	Domain × Feedback	TPJ act	SU −	-	-	<.05*
H2	Domain × Feedback	VS-dmPFC ppi	SU −	2.525	<.01**	0.04*
H2: ExA	Domain × Feedback	VS-vmPFC ppi	RS +	2.528	<.01**	0.04*

Note: ExA=First exploratory result; ExB=second exploratory result; act=activation; ppi=functional connectivity; RS=first-order measure of reward sensitivity; RS^2^=second-order measure of reward sensitivity (i.e., emphasizes aberrant RS); SU=substance use; corr-p=corrected p value.

* (p<.05)

** (p<.01).

## Data Availability

Analysis code related to this project can be found on GitHub (https://github.com/DVS-Lab/istartsocdoors). In addition, all data will be made available on OpenNeuro before publication.
