# Reward sensitivity and corticostriatal function during social rewards

**DOI:** 10.1101/2023.01.17.524305

**Published:** 2023-06-30

**Authors:** James B. Wyngaarden, Camille R. Johnston, Daniel Sazhin, Jeff B. Dennison, Ori Zaff, Dominic Fareri, Michael McCloskey, Lauren B. Alloy, David V. Smith, Johanna M. Jarcho

**Affiliations:** 1 Department of Psychology & Neuroscience, Temple University, Philadelphia, PA, USA; 2 Derner School of Psychology, Adelphi University, Garden City, NY, USA

**Keywords:** Reward Sensitivity, Social Reward Processing, Corticostriatal Connectivity

## Abstract

Aberrant levels of reward sensitivity (RS) have been linked to substance use (SU) disorder and are characterized by alterations in reward processing in the ventral striatum (VS). Less is known about how RS and subclinical SU relate to striatal function during social rewards (e.g., positive peer feedback). Testing this relation is critical towards predicting risk for development of SU disorder. In this pre-registered study, participants (N=44) underwent fMRI while completing well-matched tasks that assess neural response to reward in social and monetary domains. Contrary to our hypotheses, aberrant RS blunted the relationship between SU and striatal activation during receipt of rewards, regardless of domain. Moreover, exploratory whole-brain analyses showed unique relations between SU and social rewards in temporoparietal junction. Psychophysiological interaction (PPI) analysis demonstrated that aberrant RS is associated with increased connectivity between the VS and ventromedial prefrontal cortex during social rewards. Finally, we found that SU was associated with decreased connectivity between the VS and dorsomedial prefrontal cortex for social rewards, independent of RS. These findings demonstrate nuanced relations between RS and SU, even among those without SU disorder, and suggest altered reward-related engagement in cortico-VS as potential predictors of developing disordered behavior.

## Introduction

Trait reward sensitivity (RS) is a self-reported predisposition to seek rewarding substances and experiences (e.g., food, sex, etc.; Volkow et al., 2010). Neuroimaging studies have shown that RS relates to activity in the ventral striatum (VS) and medial prefrontal cortex (Beaver et al., 2006; Simon et al., 2010; Sripada et al., 2011; Pujara et al., 2016). Importantly, abnormalities in reward processing may underlie risky substance use (SU) (e.g., Bart et al., 2021). One theory of risk for SU disorder argues that repeated SU over time blunts reward-related activation in the VS and ventromedial prefrontal cortex (vmPFC; Beck at al., 2009; Goldstein & Volkow, 2011). Other evidence suggests SU is associated with hypersensitivity to rewards. For example, higher behavioral approach scores, which are closely linked to RS, are associated with alcohol use (Franken & Muris, 2006), cocaine addiction (Balconi & Finocchiaro, 2015), and nicotine dependence (Lawn et al., 2015). Thus, individuals with high or low (i.e., aberrant) RS are at risk for developing SU problems, irrespective of their current expression. Understanding risk for problematic SU is major health concern. Over 20 million Americans are affected by SU disorder (Avena et al., 2021), and between April 2020 and 2021, 92,000 people died from drug overdose—the highest death toll during a 12-month period on record (NCDAS, 2022). One common risk factor for developing SU disorder may be abnormalities in reward processing. For example, individuals with a hyposensitive reward system may be predisposed toward using psychoactive substances to compensate and up-regulate their reward systems (Blum et al., 1996). However, people with a hypersensitive reward system tend to be more impulsive and risk-seeking, factors that also may contribute to SU (Nusslock & Alloy, 2017). These seemingly conflicting observations suggest that disparate mechanisms may lead to a common clinical outcome. As such, it may be the case that variability in reward sensitivity can help assess the relevance of social reward processing in individuals who are not yet heavy substance users.

Adolescence and emerging adulthood are critical periods for risk/expression of SU disorder (Crews et al., 2007; Wood et al., 2018), particularly given that adolescents with aberrant RS scores are more likely to develop SU disorder (Alloy et al., 2009; Ivanov et al., 2008; Genovese & Wallace, 2007). Despite these insights, understanding of the brain-based relationship between SU and RS may be limited by a reliance on narrowly focused reward paradigms (e.g., only evaluating monetary outcomes; e.g., Dennison et al., 2022; Bart et al., 2021) performed by individuals who have already developed SU disorder. Studying diverse reward domains, including social feedback, in a population who is at risk for, but yet to express SU disorder is critical to differentiate between causal factors and consequences associated with problematic SU behaviors. If an individual is already using substances heavily, then it is hard to distinguish their current usage patterns from their future risk of having problems. Similarly, abnormal brain function may precede problematic SU, or it may emerge due to of problematic SU behavior. To this end, future efforts focused on prevention of substance use problems must target healthy individuals who are at risk for developing problems.

While SU often occurs in a social context (Goncy et al., 2013; Wei et al., 2010; Andrews et al., 2002; Dishion & Owen, 2002) and social stressors, such as peer rejection, commonly precipitate consumption (Nepon et al., 2021; Milivojevic & Sinha, 2018), relationships between SU and RS in the context of *social* rewards are surprisingly understudied. The few studies to test relations between social reward and brain function have shown that SU is associated with decreased activation in the striatum during social rewards (e.g., Jarcho et al., 2022; Zimmermann et al. 2019). However, even fewer studies include brain responses to both social and monetary rewards, which are necessary to make claims about the specificity of effects. Additionally, associations between SU and VS-based connectivity during social reward receipt remain relatively unexplored. Social rewards do elicit VS activation (Izuma et al., 2008), and recent meta-analyses show that processing social information is related to engagement of the dorsal and ventral medial prefrontal cortex (dmPFC, vmPFC), posterior cingulate cortex (PCC), right fusiform face area (FFA), and bilateral amygdala (Tso et al., 2018; Feng et al., 2021; Martins et al., 2021). Therefore, such regions may be promising targets for VS connectivity during receipt of social rewards (e.g., Sazhin et al., 2020). When assessing these relationships, controlling for trait reward sensitivity is important, as doing so helps to disentangle the specific effects of SU behaviors from individual differences in reward responsiveness.

We leveraged well-matched social and monetary reward tasks (Distefano et al., 2018; Nelson & Jarcho, 2021; Quarmley et al., 2019) to investigate associations between RS, sub-clinical SU, and brain activation and connectivity. First, we aimed to quantify the relationship between RS, SU, and VS activation during social compared to monetary rewards. Given that substance use frequently takes place in social settings and is often triggered by social stressors such as peer rejection, we hypothesized that individuals with higher levels of SU would show exaggerated VS responses to social-vs-monetary rewards, independent of RS. Second, we aimed to examine the relationship between RS, SU, and ventral striatal connectivity during social-vs-monetary rewards. We hypothesized that individuals with higher levels of SU would show enhanced connectivity between the VS and regions modulated by social information, independent of RS. By controlling for RS in both hypotheses, we can isolate the effects of SU from individual differences in RS, allowing for a more precise examination of the distinct impact of SU on neural responses to social rewards. We also conducted a series of exploratory analyses including the interaction between SU and RS to assess the nuanced impact that individual differences in trait reward sensitivity may have on the relationship between SU and social reward processing.

## Materials and Methods

### Participants

Although the pre-registration (https://aspredicted.org/blind.php?x=JNH_EGK) describes our goal to collect data from 100 participants (18–22), we ultimately studied 59 participants due to constraints imposed by the COVID-19 pandemic. Using pre-registered criteria, fifteen of the 59 participants who completed the study were excluded from analyses due either to failure to respond during behavioral tasks (>20% missing responses; N=4), incomplete data (N=5; failure to complete survey data or missing behavioral data due to technical issues), or poor image quality (N=6). Image quality was defined using the fd_mean and tSNR values from MRIQC (Esteban et al., 2017). Participants were excluded for fd_mean values greater than 1.5 times the interquartile range, or for tSNR values below the lower bound of 1.5 times the interquartile range, per the distribution from neuroimaging data of otherwise eligible participants. This resulted in a final sample of 44 participants (mean age: 20.45 years, SD: 1.89 years; 22.7% male; 57% white, 34% Asian, 9% other—2 Black/African American, 1 Black and white, 1 Indian).

Participants were recruited via the Temple University Psychology and Neuroscience Department participant pool, and from the surrounding community via flyers and online advertisements. Participants were paid $25 per hour for fMRI and $15 per hour for behavioral tasks, and received bonuses based on their decisions on other neuroeconomic tasks (not reported here), resulting in a total payment of $140 to $155. In addition, participants recruited from the university pool also received research credit for their participation.

### Procedure

All methods were approved by the Temple University IRB. Prospective participants were identified based on their responses to an online screener, which assessed RS using the Behavioral Activation Subscale (BAS; Carver & White, 1994) and the Sensitivity to Reward subscale (SR; Torrubia & Tobeña, 1984). A sum was calculated for each subscale. Sums were assigned to a quintile that reflected low to high levels of RS across the distribution of possible scores. Using methods consistent with our prior work (e.g., Alloy et al., 2009) designed to increase confidence that participants were truthful, attentive, and not responding at random, only participants with scores within +/−1 quintile on both subscales were eligible for the study (no exclusions were made based on this criteria). SU information also was collected on the online screener via the Alcohol Use Disorders Identification Test (AUDIT; Babor et al., 1992) and the Drug Use Identification Test (DUDIT; Berman et al., 2002). At the in-person visit, we confirmed that eligible participants were free of major psychiatric or neurologic illness and MRI contraindications. Breathalyzer tests, urine drug screens, and (for females) urine pregnancy tests were also collected to confirm the absence of alcohol, illicit drugs, and pregnancy prior to fMRI.

Participants were told that they would complete a social evaluation task with peers who had already completed the study. Prior to their fMRI visit, our team took a photograph of the participant that was purportedly uploaded to a study database. Participants believed that once this photograph was uploaded, peers would receive a text message on their cell phone asking them to view the photo and indicate whether they thought they would ‘like’ or ‘dislike’ the participant. Participants were told that at their fMRI visit several days later, they would be asked to guess which peers ‘liked’ and ‘disliked’ them. Participants were also told that they would be completing monetary guessing tasks.

#### fMRI-Based Tasks.

**Figure 1.**
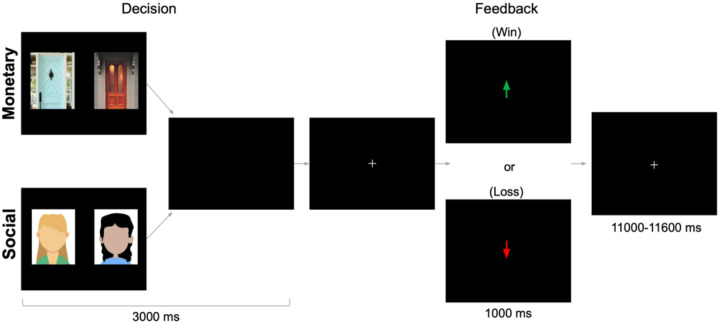
fMRI-based monetary and social reward tasks. On each trial, participants choose either between two doors (monetary task) or the faces of two peers (social task) in search of a reward. After a brief interval, they receive feedback: an upward arrow indicating a win (monetary task=$0.50 gain; social task=positive peer feedback) or a downward arrow indicating a loss (monetary task=$0.25 loss; social task=negative peer feedback).

The monetary and social reward tasks (Distefano et al., 2018; Nelson & Jarcho, 2021; Quarmley et al., 2019) were administered using PsychoPy (Peirce et al., 2019). As depicted in Figure 1, There were two conditions (monetary, social) that were presented in separate runs (order counterbalanced across participants). This design eliminates task-switching costs (Hayden et al., 2010) while still preserving our ability to directly contrast responses associated with social and nonsocial reward. In the monetary task, participants were shown two doors. Participants were instructed to pick the door that contained a $0.50 prize. On monetary reward trials, feedback indicated that the participant won $0.50. On monetary loss trials, feedback indicated that the participant lost $0.25. In the social task, participants were shown the faces of two peers. Participants were informed that each peer had indicated whether they liked or disliked the participant based on a photo of them, and they were instructed to pick the peer that liked them. On social reward trials, feedback indicated that the peer liked them. On social loss trials, feedback indicated that the peer disliked them (See supplementary materials for more details). Each run included 60 trials: 50% resulted in reward and 50% resulted in loss feedback. Although both tasks involve feedback about correct/incorrect guesses, the result of that feedback occurs in social or monetary domains. Each task collected 292 functional volumes. Trials were separated by a variable duration intertrial interval (1,100–11,600 ms; M = 3,500ms).

### Individual Difference Measures

#### Trait Reward Sensitivity.

RS was defined by a composite score consisting of the sum of the z-scores for the Behavioral Activation Scale (BAS; Carver & White, 1994) and the Sensitivity to Reward (SR) subscale of the Sensitivity to Punishment/Sensitivity to Reward Questionnaire (Torrubia et al., 2001). The BAS is a 20-item self-report questionnaire that aims to measure sensitivity to appetitive motives (e.g., “I go out of my way to get things I want”). The SR is a 24-item self-report measure aimed at more context-specific items. The total BAS and the SR subscale are reliable and valid measures of RS (Alloy et al., 2006; Alloy et al., 2012).

We tested both linear and nonlinear associations between brain responses and RS. However, assessing nonlinear associations with RS (i.e., aberrant RS) by taking the 2^nd^-order polynomial expansion overweights the tails of the distribution (Buchel et al., 1998). To avoid this, we first normalized the values by converting them to deciles, where each decile had approximately the same number of participants (cf. Winsorizing). Although this strategy was not described in our pre-registration, we believe it is a necessary deviation because it ensures that our analyses can account for both linear and nonlinear associations (e.g., U-shaped and inverted U-shaped patterns between brain responses and RS) while ensuring that results are not driven by extreme values in the tails of the distribution.

#### Substance Use.

To distinguish risk factors from consequences of problematic SU, it is crucial to study individuals who have not yet exhibited problematic SU. Current SU was defined by a composite score consisting of the sum of the z-scores for the Alcohol Use Disorders Identification Test (AUDIT; Babor et al., 1992) and the Drug Use Identification Test (DUDIT; Berman et al., 2002). The AUDIT is a 10-item self-report measure that assesses frequency (e.g., “How often do you have a drink containing alcohol?”) and disruptiveness (e.g., “How often during the last year have you failed to do what was normally expected of you because of drinking?”) of alcohol use. Scores greater than 15 are categorized as high risk for alcohol dependence (Babor et al., 2001). The DUDIT is an 11-item self-report measure that assesses frequency and disruptiveness of non-alcoholic drug use, containing references to a wide array of substances, including marijuana, cocaine, and others. Scores greater than 24 are categorized as high-risk for dependence (Berman et al., 2005). As no participant scored in the high-risk category for the AUDIT and only one participant for the DUDIT (score = 26), we characterize SU in our sample as “sub-clinical.” We summed z-scores for AUDIT and DUDIT because we did not have hypotheses differentiating between alcohol and drug use.

### Neuroimaging Data Acquisition and Preprocessing

Functional images were acquired using a 3.0 Tesla Siemens PRISMA MRI scanner and a 20-channel head coil. Neuroimaging data were converted to the Brain Imaging Data Structure (BIDS) using HeuDiConv version 0.9.0 (Halchenko et al., 2019). Results included in this manuscript were preprocessed with fMRIPrep 20.2.3 (Esteban et al., 2018a, 2018b), which is based on Nipype 1.4.2 (Gorgolewski et al., 2011, 2018). MRI acquisition and preprocessing parameters are described in the supplementary materials.

### Neuroimaging Analyses

#### Individual Level Analyses.

Neuroimaging analyses used FSL version 6.0.4 (Smith et al., 2004; Jenkinson et al., 2012). We conducted two types of analyses (activation and connectivity) to investigate how social, compared to monetary, rewards and SU were associated with BOLD responses, independent of linear or quadratic expressions of RS. Exploratory analyses also examined RS as a covariate of interest. Both used individual level general linear models with local autocorrelation (Woolrich et al., 2001).

The first model focused on the brain activation evoked during the feedback phase of each reward task (i.e., monetary and social) and used four task-based regressors. Two regressors of interest included reward and loss feedback (duration = 1,000 ms). Two regressors of no interest included the decision phase (duration = 3,000 ms) and trials with missed responses within the decision phase (i.e., failures to respond; duration = 3,000 ms).

The second model focused on task-dependent connectivity with VS as it related to the varying types of feedback in the task (i.e., reward vs. loss). To estimate the changes in connectivity between feedback types, we used psychophysiological interaction (PPI) analysis (Friston et al., 1997; O’Reilly et al., 2012), which can reveal consistent and specific task-dependent changes in connectivity (Smith et al., 2016; Smith & Delgado, 2017). We focused on feedback-dependent changes in connectivity with the bilateral VS (Oxford-GSK-Imanova atlas; Tziortzi et al., 2011). This model used a total of nine regressors. The first four were identical to those described in the activation model (i.e., reward, loss, decision, and missed trials). A fifth regressor consisted of the average timecourse of activation from the VS seed region (i.e., the physiological regressor). Four additional regressors corresponded to the interaction between the physiological regressor and each of the four original regressors.

Both activation and connectivity models included additional regressors of no interest that controlled for six motion parameters (rotations and translations), the first six aCompCor components explaining the most variance, non-steady state volumes, and framewise displacement (FD) across time. Finally, high-pass filtering (128s cut-off) was achieved using a set of discrete cosine basis functions.

### Hypothesis 1

#### ROI Group Level Analysis.

Hypothesis 1 seeks to test whether individuals reporting higher levels of SU show exaggerated ventral striatal responses to social rewards relative to monetary rewards, independent of expressions of RS. To control for potential relations with RS, both linear (i.e., greater values correspond with greater RS) and quadratic (i.e., greater values correspond with more aberrant RS) measures of RS were included as covariates of no interest. For each participant, activation in the VS seed described above was extracted using AFNI’s 3dROIstats. We used FSL’s PALM (Winkler et al., 2014; Alberton et al., 2020) to conduct a linear regression for the difference in striatal BOLD for social compared to monetary reward processing (see Table 1 for notation) that was regressed onto a model of SU including additional covariates for RS (first- and second-order measures), the SUxRS and SUxRS^2^ interactions, and nuisance regressors (tSNR and motion; seven covariates total). Lower order effects of interest were also probed in the absence of higher order interactions. Given that social and monetary tasks were administered separately, it is critical that we account for differences in the quality of confounds like temporal signal-to-noise ratio (tSNR) and framewise displacement. To account for these differences, we subtracted the value from the monetary task from the social task.

### Hypothesis 1 Exploratory Analyses

#### ROI Group Level Analysis.

We hypothesized that we would observe a domain × feedback × SU interaction. However, lower order interactions that included task effects (i.e., domain or feedback) were also explored to test the extent to which SU may relate to brain activity as a function of other task features. Exploratory ROI-based analyses also examined relations with RS as a covariate of interest.

#### Whole Brain Group Level Analyses.

We also conducted exploratory whole brain analyses to investigate regions outside of the VS that may be implicated in SU, reward, and social processes. Group-level analyses were conducted using Randomise (Winkler et al., 2014) and focused on social reward processing using the same linear regression model described above. In addition to SU, whole brain exploratory analyses also investigated relations with RS as a covariate of interest.

### Hypothesis 2

#### PPI Target Regions.

**Figure 2.**
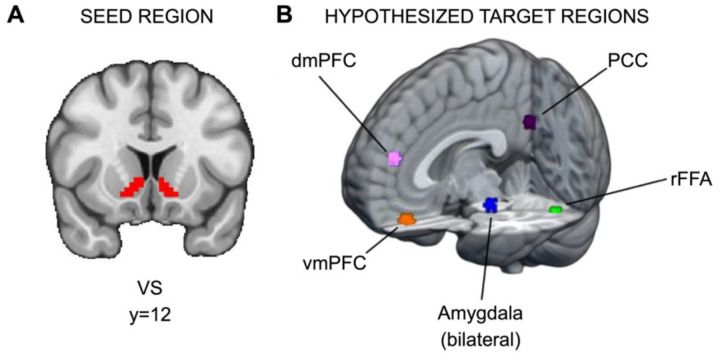
ROIs for PPI analyses. (A) Ventral striatum seed region and (B) hypothesized target regions for PPI analyses (https://identifiers.org/neurovault.collection:13364). Hypothetical target regions related to social information processing in the current dataset were identified via significant clusters in the decision phase. For each target region, a 5mm sphere was drawn around the significant cluster’s peak voxel. The amygdala targets were constrained to voxels within the amygdala as defined by Harvard-Oxford Atlas.

Hypothesis 2 seeks to investigate whether SU, independent of RS, is associated with elevated connectivity between the VS and regions implicated in social information processing (e.g., ventromedial prefrontal cortex) during responses to social, relative to monetary, reward. To this end, we sought to identify brain regions modulated by social information (i.e., stimuli in the social condition) in the current dataset. To isolate those regions, we contrasted brain function during the decision phase between the social task, where participants viewed pictures of peers, and the monetary task, where participants viewed pictures of doors. Significant clusters of activation were found in several regions, and for each potential target region, a 5mm sphere was drawn around the peak voxel in the cluster (see Figure 2 and https://identifiers.org/neurovault.collection:13364). Significant regions were the ventromedial prefrontal cortex (vmPFC; x=33, y=61, z=20), dorsomedial prefrontal cortex (dmPFC; x=34, y=62, z=34), right fusiform face area (rFFA; x=47, y=26, z=20), bilateral amygdala (right, x=39, y=42, z=22; left, x=25, y=42, z=22), and posterior cingulate cortex (PCC; x=33, y=25, z=36). There are two caveats for amygdala: first, for the right amygdala, the cluster extended across multiple regions, so a local maximum within the Harvard-Oxford-defined anatomical region for right amygdala was used instead; second, because amygdala is a smaller anatomical region relative to the other potential targets, after drawing 5mm spheres, both amygdala ROIs were constrained to the Harvard-Oxford anatomical atlas.

#### PPI ROI Analysis.

For each participant, connectivity estimates for the target regions described above (vmPFC, dmPFC, rFFA, bilateral amygdala, and PCC) were extracted using AFNI’s 3dROIstats from individual level analyses that modeled the timecourse of VS as a seed. For each target region, we used FSL’s PALM (Winkler et al., 2014; Alberton et al., 2020) to conduct linear regression models for the difference in functional connectivity between VS seed and target ROIs for social reward processing. This was regressed onto a model of SU including additional covariates for RS (first- and second-order measures), the SU × RS and SU × RS^2^ interactions, and nuisance regressors (tSNR and motion). Specifically, we hypothesized that we would observe a domain × feedback × SU interaction.

### Hypothesis 2 Exploratory Analyses

#### PPI ROI Analysis.

Hypothesis 2 examines the domain × feedback × SU interaction in the hypothesized target regions. However, lower order interactions that included task effects (i.e., domain or feedback) were also explored to test the extent to which SU may relate to connectivity as a function of other task features. In addition to SU, exploratory analyses also investigated relations with RS.

## Results

### Hypothesis 1

#### No Association between Substance Use and Ventral Striatum Activation During Social vs. Monetary Rewards.

Our first goal was to examine whether higher levels of SU were associated with exaggerated ventral striatal response to social relative to monetary rewards, independent of self-reported RS. Inconsistent with our first pre-registered hypothesis, we did not observe a significant association between SU and striatal activation for social vs. monetary rewards after controlling for RS (*t*_(36)_=−1.1077, *p*=0.865; full model results in Table 2).

### Hypothesis 1 Exploratory Results

#### Aberrant Reward Sensitivity is Associated with a Diminished Relation between Substance Use and Striatal Activation During Reward Receipt.

**Figure. 3.**
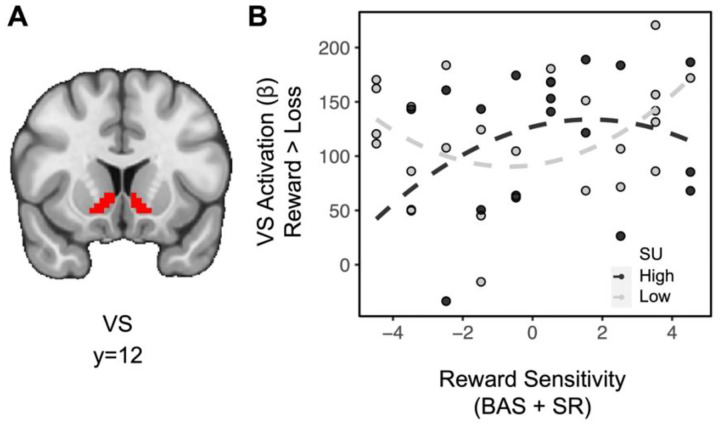
Aberrant reward sensitivity blunts the relationship between substance use and striatal activation during receipt of rewards. (A) Ventral striatum ROI. (B) For individuals with moderate levels of RS (N=26), greater levels of SU are weakly associated with striatal activation for rewards. However, for individuals with aberrant RS (N=18), greater levels of SU are associated with decreased striatal activation for rewards. [Aberrant reward was specified as individuals in the top 2 and bottom 2 deciles of the first-order RS measure (i.e., individuals with positive values for second-order RS when de-meaned)].

Exploratory analyses examining the extent to which SU and RS may relate to brain activity as a function of other task features were also conducted. In the absence of higher order interactions, lower order interactions investigating striatal effects related to domain and feedback were assessed. Across all subjects, the difference in striatal BOLD for feedback [(social reward + monetary reward) > (social loss + monetary loss)] showed a significant effect (*t*_(36)_=13.534, *p*<0.001), indicating greater striatal activation for rewards relative to losses (full model results in Table 2). Importantly, there was no difference in striatal activation between domains (e.g., *t*_(36)_=−0.774, *p*=0.779), suggesting that degree of reward processing across paradigms was similar. A linear regression for the difference in striatal BOLD for feedback on the interaction between SU and quadratic (aberrant) RS also revealed a significant effect (*t*_(36)_=2.198, *p*=0.018): among individuals with moderate trait RS, SU is weakly associated with rewarding feedback, however, among individuals with aberrant trait RS, SU is negatively associated with rewarding feedback (Fig. 3). A linear regression for the difference in striatal BOLD for domain [(social reward + social loss) > (monetary reward + monetary loss)] revealed no significant relationships.

#### Substance Use is Associated with Blunted TPJ Responses to Social vs. Monetary Reward.

**Figure 4.**
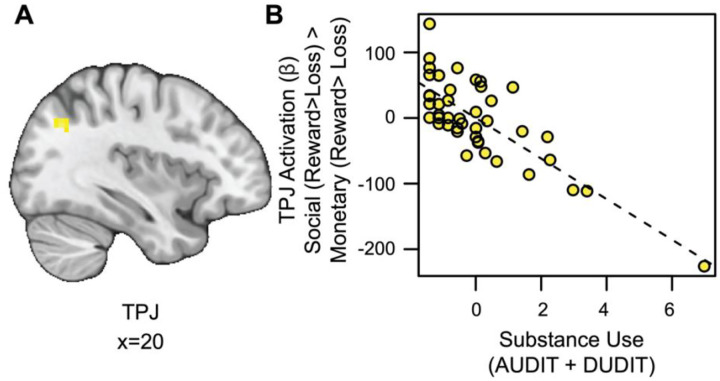
Substance use is associated with blunted TPJ responses to social vs. monetary reward. (A) Cluster-level thresholded activation (Z > 3.1, FWE = 0.05) for social rewards in the temporoparietal junction (TPJ) related to SU (Thresholded: https://identifiers.org/neurovault.image:790569; Unthresholded: https://identifiers.org/neurovault.image:790568). (B) While controlling for linear and quadratic measures of RS: as SU increases, social reward activation in the TPJ (component + residual) decreases.

We also conducted an exploratory whole-brain analysis to investigate the relation between SU and activation for social rewards beyond the VS. This analysis revealed a significant cluster of activation in the temporoparietal junction (TPJ) for the social rewards in relation to SU, after controlling for RS (see Fig. 4A and https://identifiers.org/neurovault.image:790569). Extracting parameter estimates from the TPJ (x=14, y=19, z=38; ke = 56) revealed that as SU increased, activation in the TPJ decreased for social relative to monetary reward (Fig. 4B).

### Hypothesis 2

#### Substance Use is Associated with Reduced Task-Based Connectivity between the VS and dmPFC During Social vs. Monetary Reward.

As described in our pre-registration, we hypothesized that elevated VS responses to social reward, relative to monetary reward, would be associated with enhanced connectivity with regions modulated by social information, independent of RS. To test this hypothesis, we conducted an ROI-based psychophysiological interaction (PPI) analysis using the VS as our seed and five social regions as targets. Linear regressions for lower order interactions of domain and feedback also were assessed. No significant effects were observed in the amygdala, rFFA, or PCC for our interactions of interest.

**Figure 5.**
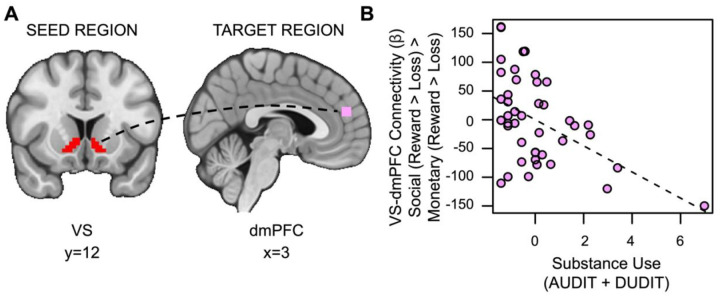
Substance use is associated with decreased VS-dmPFC connectivity for social vs. monetary reward. (A) Ventral striatum seed region and dmPFC target region. (B) As SU increases, connectivity between the ventral striatum and the dorsomedial prefrontal cortex (dmPFC) during receipt of social rewards is reduced.

Contrary to our second hypothesis, we found that VS-dmPFC connectivity for social relative to monetary reward was attenuated in individuals with more severe SU (*t*_(36)_=2.525, *p*=.007, *family-wise-error-corrected p (fwep)*=.0353; full model results in Table 3). As SU increases, connectivity between the VS and dmPFC is reduced (Fig. 5).

### Hypothesis 2 Exploratory Results

**Figure 6.**
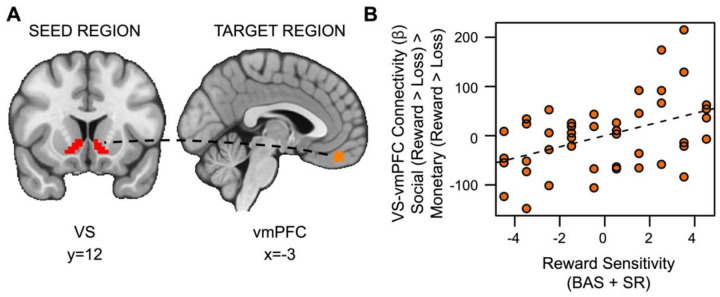
Reward sensitivity is associated with increased VS-vmPFC connectivity for social vs. monetary reward. (A) Ventral striatum seed region and vmPFC target region. (B) As reward sensitivity increases, connectivity between the ventral striatum and the ventromedial prefrontal cortex (vmPFC) during receipt of social rewards is enhanced.

Also inconsistent with our hypotheses, social rewards showed enhanced connectivity between the VS and vmPFC in relation to RS (*t*_(36)_=2.528, *p*=.007, *fwep*=.0356; full model results in Table 4). As RS increases, functional connectivity between the VS and vmPFC is enhanced (Fig. 6).

## Discussion

We leveraged well-matched social and monetary reward tasks (Distefano et al., 2018; Nelson & Jarcho, 2021; Quarmley et al., 2019) to investigate associations between RS, SU, and brain activation in young adults with a range of subclinical SU behavior. Although we hypothesized that SU would be associated with VS activation for social rewards independent of RS, we did not find support for this hypothesis. However, exploratory whole-brain analyses did find this effect in the TPJ, such that SU was associated with decreased TPJ activation for social vs. monetary rewards while controlling for RS. Moreover, exploratory investigation of lower order effects showed that aberrant RS blunts the relationship between SU and VS activation during receipt of domain-general rewards. Contrary to our second hypothesis, our findings show that SU is associated with decreased VS-dmPFC connectivity for social vs. monetary rewards. Additionally, exploratory analyses showed that RS is associated with increased VS-vmPFC connectivity for social vs. monetary rewards. Taken together, these findings suggest that sub-clinical SU is associated with blunted response (TPJ) and diminished corticostriatal connectivity (VS-dmPFC) for social relative to monetary rewards, while underscoring the nuanced role of trait RS. Moreover, our results help disentangle results from previous studies that focus on participants with SU disorder, which make it difficult to determine if altered relations are a cause or consequence of long-term disordered behavior.

Very few studies have investigated the impact of sub-clinical SU on processing social rewards (see Beard et al., 2022). However, our finding that SU is associated with blunted brain activation (TPJ) and connectivity (VS-dmPFC) for social compared to monetary rewards is in line with the handful of studies. For example, Zimmermann et al. (2019) found that cannabis users showed less activity in the striatum during interpersonal touch from a female experimenter, while non-users showed more striatal activity. Further, Li et al. (2021) showed that binge drinkers had decreased neural responses in the anterior medial orbitofrontal cortex and precuneus when viewing social interactions of abstract shapes, compared to viewing random movement. Finally, a recent study from our research group (Jarcho et al., 2022), completed after preregistration for the current work, found that greater substance abuse behavior in adolescents was related to decreased right VS response to social rewards (peer ‘like’ feedback). Given that SU often occurs in a social context (Goncy et al., 2013; Wei et al., 2010; Andrews et al., 2002; Dishion & Owen, 2002) and social stressors commonly precipitate consumption (Nepon et al., 2021; Milivojevic & Sinha, 2018), these domain-specific effects are worth investigating further.

Our findings also provide evidence suggesting that SU and RS are associated with altered connectivity in regions that support social cognition. SU was related to decreased VS-dmPFC connectivity for social rewards, potentially suggesting that increased SU is related to altered ability to process social information. Striatal connectivity has been linked to interpreting others’ facial expressions (Kim et al., 2020), and the dmPFC has been implicated in social cognitive functions during receipt of social feedback (e.g., Ferrari et al., 2016) and prediction error during social learning (Joiner et al., 2017; Suzuki et al., 2012). Reduced correspondence of the VS with dmPFC during social reward may suggest an impaired facility for assessing and reacting to feedback from others. Additionally, contrary to our hypothesis, trait RS was positively associated with VS-vmPFC connectivity for social rewards. Increased subjective utility for a given choice after observing others make a similar choice has been linked to the vmPFC (Chung et al., 2015), and functional connectivity between the VS and the vmPFC has been shown to reflect subjective value for social rewards (Smith et al., 2014; Hill et al., 2017; Hare et al., 2010). The current results suggest that higher trait RS is associated with increased functional integration of these regions, perhaps bridging the gap to previous research linking RS to social functions like extraversion (Lucas et al., 2000) and anxiety (Booth & Hasking, 2009) which may center on receiving feedback from peers.

There are several future directions to consider when evaluating these results. A benefit of studying those at risk for SU is that effects observed here are unlikely to be a consequence of long-term use that often occurs in clinical populations. Previous research has shown the association between responses in the nucleus accumbens/VS during reward anticipation and future problematic substance use and relapse (e.g., Büchel et al., 2017; MacNiven et al., 2018). However, while the limited range of SU in our sample allows us to examine risk factors for SU disorder, the subtle effects we observe here may differ from observations of a clinical sample. Moreover, wider variability in SU may help distinguish between the effects of different types of substances (e.g., alcohol vs. marijuana) that may occur in different settings and have more nuanced relationships with social context and reward sensitivity. To help close the gap between cause and consequence, longitudinal studies could be used to further examine the relationship between sub-clinical SU and development of SU disorder. Other features of the sample may impact generalizability. For example, the current sample is comprised of college students, who have lower levels of SUD relative to their non-college peers (despite higher rates of heavy alcohol consumption; Skidmore et al., 2016). Differences in social factors related to SU for college vs. non-college individuals may be relevant to consider as well. Finally, the sample is also relatively small, primarily white (57%) and Asian (34%), and predominantly female (77.3%). SUD is more common in males (Brady & Randall, 1999), although this gap is narrowing (Steingrimsson et al., 2012). As neuroimaging research on sex-based differences in relation to substance use and the brain is mixed (e.g., McHugh et al., 2018), further work is needed in larger samples to test for these important biological differences.

Despite these limitations, the present results demonstrate that varying levels of problematic SU in a sub-clinical population is associated with variations in activation and task-based connectivity in regions implicated in social processing while experiencing social rewards. Moreover, we show that trait RS is an important factor when individuals experience social feedback. Although SUD is a complicated issue, our findings help characterize the important roles in how social factors and aberrant reward sensitivity are related to problematic SU. These results may contribute to our understanding of how to identify and reduce instances of SUD in the future.

## Supplementary Material

Supplement 1

Supplement 2

## Figures and Tables

**Table 1.** Terms for model contrasts.

Term	Effect	Contrast
Social reward processing	Domain × Feedback	Social (Reward > Loss) > Monetary (Reward > Loss)
Reward processing	Feedback	(Social Reward + Monetary Reward) > (Social Loss + Monetary Loss)
Social processing	Domain	(Social Reward + Social Loss) > (Monetary Reward + Monetary Loss)
Decision phase	Social Stimuli	(Social Decision > Baseline) > (Monetary Decision > Baseline)

**Table 2.** Statistics for regression models of striatal activation.

	Domain × Feedback		Domain		Feedback	
Contrast	*tstat*	*uncp*	*tstat*	*uncp*	*tstat*	*uncp*
Main Effect +	−0.7739	0.7793	0.6244	0.257	13.5344	0.0002***
Main Effect −	0.7739	0.2208	−0.6244	0.7431	−13.5344	0.9999
RS +	−0.1752	0.5725	0.5333	0.2985	1.542	0.0619
RS −	0.1752	0.4276	−0.5333	0.7016	−1.542	0.9382
RS^2^ +	−1.4749	0.9226	0.0187	0.4916	0.3478	0.3601
RS^2^ −	1.4749	0.0775	−0.0187	0.5085	−0.3478	0.64
SU +	−1.1077	0.8649	0.3464	0.3596	−1.1312	0.8693
SU −	1.1077	0.1352	−0.3464	0.6405	1.1312	0.1308
SU × RS +	−0.283	0.6148	−0.0643	0.5298	−0.2062	0.5794
SU × RS −	0.283	0.3853	0.0643	0.4703	0.2062	0.4207
SU × RS^2^ +	0.4043	0.3447	−0.611	0.7281	−2.198	0.9817
SU × RS^2^ −	−0.4043	0.6554	0.611	0.272	2.198	0.0184*

Note: RS=first-order measure of reward sensitivity; RS^2^=second-order measure of reward sensitivity (i.e., emphasizes aberrant RS); SU=substance use.

+=positive relationship with striatal activation; −=negative relationship with striatal activation

* (p<.05);

** (p<.01);

*** (p<.001).

**Table 3.** Statistics for regression models of VS connectivity with dmPFC.

	Domain × Feedback			Domain		
Contrast	*tstat*	*uncp*	*fwep*	*tstat*	*uncp*	*fwep*
Main Effect +	0.4504	0.32	0.7291	1.1788	0.1249	0.4363
Main Effect −	−0.4504	0.6801	0.9672	−1.1788	0.8752	0.9999
RS +	1.7279	0.0467*	0.1757	−0.0671	0.5388	0.959
RS −	−1.7279	0.9534	0.9999	0.0671	0.4613	0.9367
RS^2^ +	−0.3105	0.6242	0.9563	−0.5649	0.7199	0.9929
RS^2^ −	0.3105	0.3759	0.7923	0.5649	0.2802	0.7646
SU +	−2.5251	0.9931	1	0.392	0.3437	0.8307
SU −	2.5251	0.007**	0.0353*	−0.392	0.6564	0.9836
SU × RS +	2.0168	0.025*	0.1017	0.9121	0.1826	0.6021
SU × RS −	−2.0168	0.9751	1	−0.9121	0.8175	0.9993
SU × RS^2^ +	−0.2995	0.6126	0.9521	0.786	0.2162	0.6743
SU × RS^2^ −	0.2995	0.3875	0.801	−0.786	0.7839	0.9984

	Feedback		
Contrast	*tstat*	*uncp*	*fwep*
Main Effect +	−0.2658	0.5976	0.922
Main Effect −	0.2658	0.4025	0.7813
RS +	−0.9607	0.8216	0.9924
RS −	0.9607	0.1785	0.4735
RS^2^ +	−0.1298	0.5476	0.898
RS^2^ −	0.1298	0.4525	0.8316
SU +	2.1169	0.0215*	0.0868
SU −	−2.1169	0.9786	1
SU × RS +	0.4564	0.323	0.7135
SU × RS −	−0.4564	0.6771	0.9586
SU × RS^2^ +	0.6934	0.2458	0.5965
SU × RS^2^ −	−0.6934	0.7543	0.9783

Note: RS=first-order measure of reward sensitivity; RS^2^=second-order measure of reward sensitivity (i.e., emphasizes aberrant RS); SU=substance use.

+=positive relationship with VS-dmPFC connectivity; −=negative relationship with VS-dmPFC connectivity

* (p<.05);

** (p<.01);

*** (p<.001).

**Table 4.** Statistics for regression models of VS connectivity with vmPFC.

	Domain × Feedback			Domain		
Contrast	*tstat*	*uncp*	*fwep*	*tstat*	*uncp*	*fwep*
Main Effect +	0.0052	0.5048	0.8795	1.8072	0.0389*	0.1716
Main Effect −	−0.0052	0.4953	0.8891	−1.8072	0.9612	1
RS +	2.5282	0.0074**	0.0356*	0.6809	0.2502	0.7327
RS −	−2.5282	0.9927	1	−0.6809	0.7499	0.9968
RS^2^ +	0.8768	0.192	0.5382	−1.7859	0.9597	1
RS^2^ −	−0.8768	0.8081	0.9943	1.7859	0.0404*	0.1827
SU +	−0.5602	0.7031	0.979	2.2154	0.0153*	0.0778
SU −	0.5602	0.297	0.7057	−2.2154	0.9848	1
SU × RS +	2.1603	0.019*	0.0766	1.463	0.0758	0.3075
SU × RS −	−2.1603	0.9811	1	−1.463	0.9243	1
SU × RS^2^ +	0.8184	0.2105	0.57	−0.4081	0.6579	0.991
SU × RS^2^ −	−0.8184	0.7896	0.9929	0.4081	0.3422	0.8391

	Feedback		
Contrast	*tstat*	*uncp*	*fwep*
Main Effect +	−0.1127	0.5431	0.8886
Main Effect −	0.1127	0.457	0.8338
RS +	0.4358	0.3319	0.7108
RS −	−0.4358	0.6682	0.9587
RS^2^ +	0.9296	0.1818	0.4796
RS^2^ −	−0.9296	0.8183	0.9895
SU +	−0.0513	0.5201	0.8856
SU −	0.0513	0.48	0.8556
SU × RS +	−2.2799	0.9847	1
SU × RS −	2.2799	0.0154*	0.0661
SU × RS^2^ +	0.0171	0.4936	0.8672
SU × RS^2^ −	−0.0171	0.5065	0.8705

Note: RS=first-order measure of reward sensitivity; RS^2^=second-order measure of reward sensitivity (i.e., emphasizes aberrant RS); SU=substance use.

+=positive relationship with VS-vmPFC connectivity; −=negative relationship with VS-vmPFC connectivity

* (p<.05);

** (p<.01);

*** (p<.001).

**Table 5.** Summary of reported results.

Hyp	Analysis	Effect	Contrast	*tstat*	*p*	*corr-p*
H1	Domain × Feedback	VS act	SU +	−1.108	0.87	-
H1: ExA	Feedback	VS act	SU × RS^2^ −	2.198	<.05*	-
H1: ExB	Domain × Feedback	TPJ act	SU −	-	-	<.05*
H2	Domain × Feedback	VS-dmPFC ppi	SU −	2.525	<01**	0.04*
H2: ExA	Domain × Feedback	VS-vmPFC ppi	RS +	2.528	<01**	0.04*

Note: ExA=First exploratory result; ExB=second exploratory result; act=activation; ppi=functional connectivity; RS=first-order measure of reward sensitivity; RS^2^=second-order measure of reward sensitivity (i.e., emphasizes aberrant RS); SU=substance use; corrp=corrected p value.

* (p<.05);

** (p<.01).

## Data Availability

Analysis code related to this project can be found on GitHub (https://github.com/DVS-Lab/istart-socdoors). In addition, all data will be made available on OpenNeuro before publication.
