# Supplementary material for: Reward sensitivity and corticostriatal function during social rewards
Source: bioRxiv. 2023 Jun 30:2023.01.17.524305. Originally published 2023 Jan 19. Preprint. [Version 2] doi: 10.1101/2023.01.17.524305 (PMC9882176; doi:10.1101/2023.01.17.524305)
Supplement: Supplementary file 1 [file media-1.docx]

**Supplemental Methods 1. Monetary and Social fMRI-based Tasks**

**Monetary Reward Task.** Participants were instructed to choose the door behind which there was a $0.50 prize, and were told that if they chose incorrectly, they would receive a $0.25 loss. Each trial began with the presentation of two doors. Participants used a button box to select either the left or right door on the screen. This “decision phase” totaled 3,000 ms. Specifically, when a button was pressed, a white border appeared around the selected door for 500 ms before stimulus offset. After stimulus offset, a blank screen appeared for the remainder of the 3,000 ms decision phase. A fixation cross then was presented (540 ms) before participants received feedback about the accuracy of their choice (1,000 ms). On reward trials, feedback was a green arrow pointing upwards, meaning the participant correctly selected the winning door. On loss trials, feedback was a red arrow pointing downwards, meaning the participant incorrectly selected the losing door. On trials where the participant failed to respond, feedback was randomly selected. Note that the first 12 participants, collected prior to the pandemic, saw pairs of fractals during this task. After data collection resumed, pictures of doors were used instead to more closely match with the realistic features of peer images. This change did not impact response time following monetary rewards vs. losses (Welch Two Sample t-test; t = 0.75, p = 0.46) nor striatal response to monetary rewards vs. losses (Welch Two Sample t-test; t = 0.13, p = 0.90). We therefore collapsed across participants with fractal vs. door stimuli. 

**Social Reward Task.** The social task was identical to the monetary task, except images of gender-matched (i.e., two female faces or two male faces) and age-matched peers were presented instead of doors. The social reward task consisted of 120 images compiled from multiple sources (internet databases of non-copyrighted images of college-aged individuals). The pictures of purported peers had positive facial expressions, were cropped so that individuals were pictured from their shoulders up and were edited to have an identical solid gray background. Smiling faces were used because they are common in social reward tasks (Richards et al., 2013; Jarcho et al., 2015; Distefano et al., 2018), and are subject to less misinterpretation than neutral faces (Rapee and Heimberg, 1997; Davis et al., 2016). Images were constrained to a standard size (aspect ratio: 11.2 width, 17.14 height). There were an equal number of trials with male and female peers across the reward and loss conditions (30 pairs each, 60 total). Participants were instructed to choose the peer that liked them based on the photograph of the participant. On reward trials, feedback was a green arrow pointing upwards, meaning the participant correctly selected the person who said they would like the participant. On loss trials, feedback was a red arrow pointing downwards, meaning they incorrectly selected the person who said they would dislike the participant. On trials where the participant failed to respond, feedback was randomly selected.
